# The *Aspergillus* Genome Database: multispecies curation and incorporation of RNA-Seq data to improve structural gene annotations

**DOI:** 10.1093/nar/gkt1029

**Published:** 2013-11-03

**Authors:** Gustavo C. Cerqueira, Martha B. Arnaud, Diane O. Inglis, Marek S. Skrzypek, Gail Binkley, Matt Simison, Stuart R. Miyasato, Jonathan Binkley, Joshua Orvis, Prachi Shah, Farrell Wymore, Gavin Sherlock, Jennifer R. Wortman

**Affiliations:** ^1^Broad Institute of Harvard and MIT, 7 Cambridge Center, Cambridge, MA 02141, USA ^2^Department of Genetics, Stanford University Medical School, Stanford, CA 94305-5120, USA and ^3^Institute for Genome Sciences, University of Maryland School of Medicine, Baltimore, MD 21201, USA

## Abstract

The *Aspergillus* Genome Database (AspGD; http://www.aspgd.org) is a freely available web-based resource that was designed for *Aspergillus* researchers and is also a valuable source of information for the entire fungal research community. In addition to being a repository and central point of access to genome, transcriptome and polymorphism data, AspGD hosts a comprehensive comparative genomics toolbox that facilitates the exploration of precomputed orthologs among the 20 currently available *Aspergillus* genomes. AspGD curators perform gene product annotation based on review of the literature for four key *Aspergillus* species: *Aspergillus nidulans*, *Aspergillus oryzae*, *Aspergillus fumigatus* and *Aspergillus niger*. We have iteratively improved the structural annotation of *Aspergillus* genomes through the analysis of publicly available transcription data, mostly expressed sequenced tags, as described in a previous NAR Database article (Arnaud *et al.* 2012). In this update, we report substantive structural annotation improvements for *A. nidulans*, *A. oryzae* and *A. fumigatus* genomes based on recently available RNA-Seq data. Over 26 000 loci were updated across these species; although those primarily comprise the addition and extension of untranslated regions (UTRs), the new analysis also enabled over 1000 modifications affecting the coding sequence of genes in each target genome.

## INTRODUCTION

The *Aspergillus* Genome Database (AspGD; http://www.aspgd.org/) is a web-accessible resource that collects genome sequences of the aspergilli and performs genome annotation, comparative genomics and curation of the experimental literature. The aspergilli are a diverse group of fungi that include a model genetic organism, *A. nidulans* (1), an important pathogen of immunocompromised individuals, *A. fumigatus* (2), agriculturally important toxin producers, *Aspergillus flavus* and *Aspergillus parasiticus* (3) and species used extensively in industrial processes, *A. niger* and *A. oryzae* (4,5). Evolutionarily, the aspergilli are distant enough from each other that most genes and genomic regions show significant divergence; however, they are close enough that orthologs can be identified for the majority of genes, and syntenic regions can be aligned between the genomes. The ability to align the sequences and annotations of multiple genomes leverages the power of comparative genomics and facilitates the identification and analysis of novel or important genomic features, such as secondary metabolite biosynthetic gene clusters, which are common in the aspergilli.

AspGD provides visualization tools for genomic features and alignments as well as a comparative genomics toolbox for identifying and browsing ortholog clusters and syntenic regions. Additionally, AspGD is committed to the maintenance and improvement of the structural (primary) annotation, performing iterative improvement of gene models across the aspergilli by incorporating new data and cutting edge analysis approaches. AspGD also performs expert curation of the *Aspergillus* literature to update the functional (secondary) annotation for genes in these species, comprehensively collecting gene names and phenotypes, assigning Gene Ontology (GO) terms, writing concise gene descriptions and linking all of these attributes back to the literature.

## EXPANSION OF THE NUMBER OF GENOMES HOSTED BY AspGD

During the past year, the number of genomes hosted by AspGD has doubled (Table 1), and AspGD now includes 10 additional species that were recently sequenced and annotated by the Joint Genome Institute. With this expansion, our comparative genomics toolbox, which is based on the Sybil platform (6), now hosts 16 351 clusters of orthologous genes (COGs) shared by at least two species. Among those, 3199 comprise orthologs conserved across all 20 species in AspGD (Table 1). The reduction in the number of conserved COGs after the inclusion of the 10 additional species (from 5263 to 3199) (Table 1) is due to a combination of factors: variable quality of the genome sequences, distinct methods of annotation used on each genome and the presence of distantly related species among the novel genomes. Genome statistics (Table 1) computed by the Sybil comparative platform are available at the AspGD website. Sybil can also compute the distribution of clusters across any combination of species and provides, among other functionalities, a graphical overview of the genomic context where each ortholog member is located.

**Table 1.** Incorporation of genomes into AspGD

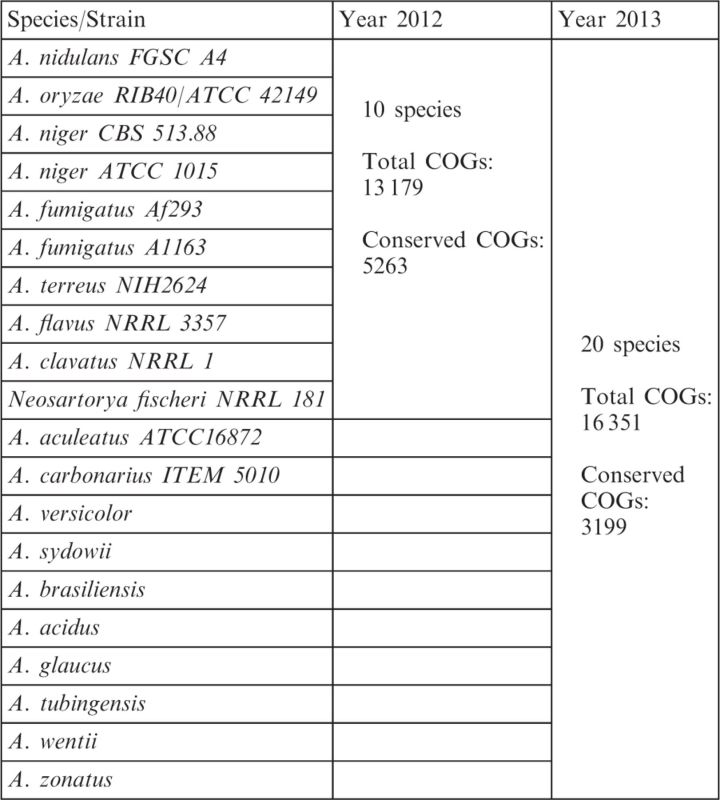

Statistics regarding COGs: total number of clusters and number of clusters conserved across all species hosted in AspGD.

A full description of the source of the sequence and the gene model modifications applied to each genome hosted by AspGD is available at http://www.aspergillusgenome.org/help/SequenceHelp.shtml. In addition, we provide a summary of genome versions for the four species for which we actively perform literature curation at http://www.aspergillusgenome.org/cgi-bin/genomeVersionHistory.pl.

## ANNOTATION IMPROVEMENT

The correct structural annotation of genes is critical to downstream functional genomics approaches. Genes that are missed by gene prediction algorithms, incorrect gene boundaries, misplaced or missing exons and wrongly merged genes can jeopardize attempts to produce an accurate catalog of metabolic potential or develop experimental probes. Transcript evidence in the form of expressed sequence tags (ESTs) or RNA-Seq data can be used to improve the structural annotation of previously annotated genomes. We are currently leveraging the wealth of recently generated RNA-Seq data to comprehensively update *Aspergillus* gene structures in a streamlined and automated fashion. Our approach consists of assembling partial transcript sequences from RNA-Seq data using the assembler Trinity (7), then aligning the resulting transcript assemblies to their respective genomic loci and updating gene models based on the new transcript evidence using the PASA pipeline (Program to Assemble Spliced Alignments) (8).

We have generated improved structural annotation for *A. oryzae RIB40*, *A. nidulans FGSC A4* and *A. fumigatus* strains *Af293* and *A1163* (Table 2). The updated gene models were based on RNA-Seq data that were either publicly available (9,10) (J. Craig Venter Institute, NCBI-SRA project number: SRP003796) or directly provided by collaborators Dr Kazuhiro Iwashita (National Research Institute of Brewing, Hiroshima, Japan) and Dr Mark Caddick (School of Biological Sciences, University of Liverpool, Liverpool, UK). The RNA-Seq data derived from strains ku80d and A1163 of *A. fumigatus* was used in combination to improve the structural annotation of each strain in AspGD: *A. fumigatus* A1163 and Af293. Only assembled transcripts with 95% identity to the reference genome were used.

**Table 2.** Statistics of gene model updates

Number of genes updated by each modification type	*A. nidulans*	*A. oryzae*	*A. fumigatus* Af293	*A. fumigatus* A1163
Total number of genes in the genome	10 982	12 176	10 073	10 106
Total number of updated genes	7729 (70%)	8390 (69%)	5183 (51%)	4854 (48%)
Merged genes	36 (now 18)	284 (now 138)	28 (now 14)	36 (now 18)
Altered coding sequence	1340 (12%)	1930 (16%)	1685 (17%)	1422 (14%)
Extended 5′UTR	7043 (64%)	7125 (59%)	4534 (45%)	4201 (42%)
Extended 3′UTR	7289 (66%)	6336 (52%)	3548 (35%)	3560 (35%)
Terminal exons added	750 (7%)	1182 (10%)	1255 (12%)	951 (9%)
Introns added or modified	904 (8%)	1188 (10%)	1133 (11%)	919 (9%)

Percentages are relative to the total number of genes in the genome of each species or strain.

We made transcription-supported modifications to 48–70% of all genes in each genome analyzed (Table 2). The most frequent change consisted of the addition or extension of 5′ and 3′ UTRs: approximately 7000 genes had modifications of this type in *A. nidulans* and *A. oryzae* and ∼5000 in the *A. fumigatus* strains. The predominance of this type of modification in *A. fumigatus* was expected given that UTRs were not yet defined for gene models in *A. fumigatus* strains. We had previously used a similar approach to annotate UTRs in *A. nidulans, A. oryzae* and *A. niger*, but that work was solely based on expressed sequence tags (ESTs) as the underlying experimental data (11).These previous EST-based modifications were predominantly restricted to highly expressed genes, as the EST approach is much less sensitive than RNA-Seq in the detection of lower-abundance transcripts.

**Figure 1.**
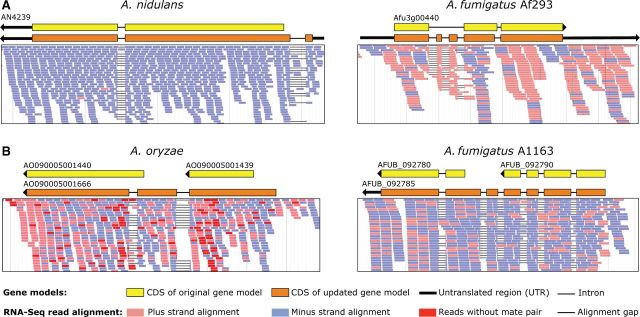
Examples of gene structural modifications supported by RNA-Seq data for *A. nidulans*, *A. oryzae* and *A. fumigatus* genomes. (**A**) Gene models with new exons added based on transcription evidence. (**B**) Gene models that were merged based on RNA-Seq evidence. Colored horizontal bars represent either gene features or RNA-Seq read alignments as described in the legend. A strand-specific RNA-Seq data set is shown in the example featuring *A. nidulans* gene AN4239.

Each updated species had >1000 loci that were subject to modifications in the coding sequence (two examples shown in Figure 1A) and dozens of genes were merged based on supporting transcription evidence (two examples shown in Figure 1B). Surprisingly, we found 8- to 10-fold more cases of genes incorrectly fragmented in *A. oryzae* compared with the other species, and we merged these fragmented genes as part of this annotation effort (Table 2). The inflated number in *A. oryzae* cannot be explained by higher RNA-Seq read coverage for this species (182× coverage for *A. oryzae*, 111× for *A. nidulans*, 353× for *A. fumigatus* Af293 and 345× for *A. fumigatus* A1163), suggesting that this effect is possibly because of gene fragmentation resulting from systematic biases during the original annotation of this genome.

The gene model updates described here were incorporated into the current version of each respective reference genome annotation available through AspGD. We are currently assessing potential novel gene annotations supported by RNA-Seq data, and defining the criteria by which RNA-Seq data provide for support of novel transcripts, and we plan to add these new genes to the data sets in the future. We will also continue to incorporate RNA-Seq data into additional *Aspergillus* genomes as these data become available.

## MULTISPECIES CURATION

AspGD literature curation began with a single species, *A. nidulans*, but we have now expanded the curation effort to routinely collect, read and extract information from all of the pertinent articles published on *A. nidulans, A. fumigatus, A. niger and A. oryzae.* In consultation with the community, we selected *A. nidulans* as the first species for curation because it serves as a well-characterized genetic model for the aspergilli and has the greatest amount of published experimental literature. We use community guidance to prioritize new species for literature curation.

The curation pipeline makes use of automation where possible, but remains a fundamentally manual time-intensive process performed by scientific curators with expertise in fungal biology. For each species, we systematically review the published literature, connecting gene names to genes, determining GO annotations, recording mutant phenotypes and writing short free-text descriptions for characterized genes. We query PubMed automatically on a weekly basis for relevant publications and prioritize the articles that contain gene-specific information. We have curated the entire corpus of gene-focused literature *for A. nidulans, A. fumigatus, A. niger* and *A. oryzae*, and have made every phenotype and GO annotation currently possible for these species, based on the available published experimental data. In total, we have curated gene-specific information from over 3000 articles. The publication rate for *Aspergillus* relevant articles showed a distinct jump following the release of the first *Aspergillus* sequences. There are now about 200 relevant articles published per year, which is roughly double the number that there was a decade ago.

In addition to maintaining and updating the curation of gene-focused data from the latest research articles, we design and undertake more specialized projects to improve the information available to the scientific community. We recently completed a targeted curation effort to overhaul the annotation of genes involved in secondary metabolism (12), which is not only an important biological process in the aspergilli but is also of particular clinical significance, as toxic secondary metabolites are known to be expressed *in vivo* during infection. As part of that curation project, we made sweeping improvements to the Biological Process branch of the GO, added hundreds of new GO terms and then used these terms to improve the breadth and specificity of *Aspergillus* annotations for proteins involved in secondary metabolic processes.

To supplement our literature-based functional gene annotations, we have developed a pipeline that infers GO annotations from experimentally characterized orthologs in *Saccharomyces cerevisiae*, *Schizosaccharomyces pombe*, *Neuorospora crassa*, *Candida albicans* and between the curated *Aspergillus* species in AspGD to uncharacterized *Aspergillus* genes. InterPro protein domains and motifs (13) are also used to make additional GO predictions. We currently provide almost 100 000 of these orthology and domain-based GO annotations across the four species that we currently curate. Many of the genes for which we infer annotations are unlikely to be characterized directly in all species, and thus our rapid and automated pipeline allows us to provide the most relevant and up-to-date predicted annotations possible.

## WEB SITE ENHANCEMENTS

**Figure 2.**
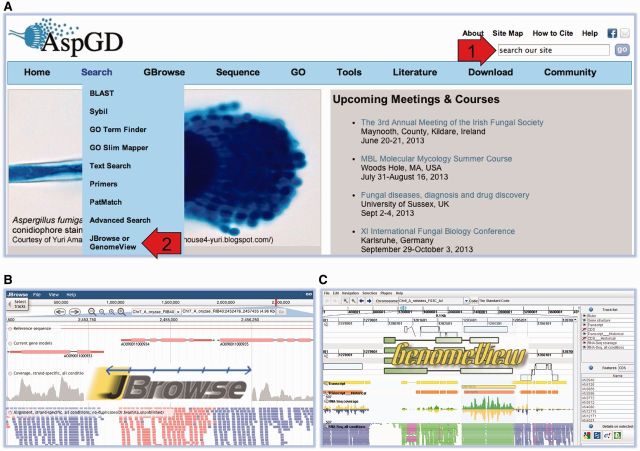
Enhancements to the website navigation and integration with JBrowse and GenomeView genome browsers. (**A**) New look and feel of AspGD user interface with updated navigation bar. (**B**) JBrowse instance depicting genes and RNA-Seq reads aligned to *A. oryzae* chromosome 7: red and blue rectangles on the bottom track indicate reads aligned to the plus and minus strand, respectively. (**C**) GenomeView instance showing the genomic context of *A. nidulans* gene AN11070. The RNA-Seq aligned reads are represented by green (plus strand) and blue (minus strand) horizontal bars in the bottom panel. Pink horizontal bars indicate alignment gaps across intronic regions.

Recently we have also undertaken several major projects to improve the ease and rapidity with which our users can obtain the data that they need. We overhauled and modernized the entire AspGD user interface. We based this project on the Web site improvements recently made at the *Saccharomyces* Genome Database (SGD) (14), which allowed us to make these changes efficiently, with maximal reuse of existing software and minimal duplication of effort. Because AspGD was originally based on the SGD framework, and many AspGD users are also long-time users of SGD, keeping the interfaces in sync with each other makes it easy for someone who is familiar with one database to quickly learn to navigate the other. The user interface overhaul includes new and improved navigation options and a quick-link menu bar, a streamlined and modernized home page with an at-a-glance listing of upcoming meetings of interest (Figure 2A) and more sophisticated search functionality. The Quick Search box (Figure 2A, arrow number 1), which is present on every AspGD web page, now features an autocomplete function. As text is typed into the search box, indexed suggestions from the database appear on a drop-down menu, allowing users to more quickly find the information they need.

In a major expansion of the data we make available, we have deployed two genome viewers, which allow users to search, browse and visualize large-scale sequencing data, such as alignments of RNA-Seq and genome resequencing data. Our primary large-scale dataset viewer is JBrowse (15), a stable and responsive open-source Javascript-based genome viewer. It seamlessly supports most web browsers and can use multiple types of data in a variety of common genomic data formats. JBrowse instances can be started from any Locus Summary page in AspGD, and the application will automatically pan to the genomic context of the locus of interest. We also offer the GenomeView (16) (Figure 2C) genome browser as a second alternative. This open-source application is based on Java and, as such, is not as widely browser compatible. Despite that, GenomeView is a full-featured genome browser with additional capabilities and customizations not available in JBrowse. GenomeView instances can be started through the drop-down ‘Search’ menu on AspGD home page (Figure 2A, arrow number 2).

## COMMUNITY INTERACTION

As a community-focused resource, we foster a strong and collaborative relationship with the researchers who use AspGD. We consult with the community regularly at conferences such as the International *Aspergillus* Meeting (Asperfest) and Advances Against Aspergillosis, as well as more broad-based fungal conferences such as the Fungal Genetics Conference and European Conference on Fungal Genetics. We encourage users to contact us by email (at aspergillus-curator@lists.stanford.edu) with questions or suggestions, and we respond promptly.

## FUNDING

Funding for open access charge: National Institute of Allergy and Infectious Diseases at the US National Institutes of Health [R01 AI077599 to G.S. and J.W.].

*Conflict of interest statement*. None declared.
